# Characteristics and postoperative dynamic changes in circulating CD4^+^ helper T lymphocytes in patients with breast cancer

**DOI:** 10.3389/fonc.2023.1118346

**Published:** 2023-02-28

**Authors:** Yan Lu, Qiaohong Zhang, Jiang Wang, Longyi Zhang

**Affiliations:** ^1^ Clinical Laboratory, DongYang People’s Hospital, Dongyang, Zhejiang, China; ^2^ Department of Breast Surgery, DongYang People’s Hospital, Dongyang, Zhejiang, China

**Keywords:** CD4^+^ helper T cell, breast cancer, Immunity, Immunosuppression, surgical resection

## Abstract

**Introduction:**

Circulating CD4^+^ helper T cell (Th) subsets provide potentially important information on disease progression in several cancers. In this study, we explored the characteristics and postoperative dynamic changes in circulating CD4^+^Th subsets in patients with breast cancer.

**Methods:**

Circulating CD4^+^Th subsets, including CD4^+^ naive T cells (Tn), CD4^+^ central memory T cells (Tcm), CD4^+^ effector memory T cells (Tem), CD4^+^CD57^+^T, and CD4^+^PD-1^+^T, were detected with multiparameter flow cytometry. T-test and Wilcoxon rank-sum test were used to compare differences between groups for normally and non-normally distributed continuous variables, respectively. Postoperative dynamic changes in CD4^+^Th subsets were assessed using the paired-sample rank-sum test.

**Results:**

Seventy-five patients with invasive breast cancer and fifty-three patients with benign breast tumors were enrolled. Compared with that in patients with benign tumors, the proportion of CD4^+^Tn in patients with breast cancer patients decreased, whereas the proportion and absolute number of CD4^+^CD57^+^T and CD4^+^PD-1^+^T increased. Moreover, the proportion of CD4^+^PD-1^+^T was correlated with the clinicopathology of breast cancer. After tumor resection, the proportion and absolute number of CD4^+^Tcm significantly decreased, while those of CD4^+^Tem significantly increased, compared with preoperative values. Tumor resection caused significant changes in the proportion and absolute number of CD4^+^CD57^+^T and CD4^+^PD-1^+^ T, both of which showed significant decreases.

**Discussion:**

We found significant changes in circulating CD4^+^Th subsets in patients with breast cancer. Additionally, complete tumor resection can benefit the patient as it balances the patient’s immunosuppression and immune stress and improves the immune exhaustion and immunosenescence states.

## 1 Introduction

Globally, breast cancer poses a serious risk to women’s health (1). It is well-established that immunosuppression and immune dysfunction cause malignant tumors to develop and spread (2). T cell subsets play an important role in cellular immunity and are being studied as possible targets for clinical biomarkers and cancer treatment (3, 4). Various T cell subsets have different functions in activating or inhibiting antitumor immune responses. Studies on antitumor immunity have mostly focused on CD8^+^ cytotoxic T cells (Tc) while paying little attention to CD4^+^ helper T (Th) lymphocytes. However, it is well known that CD4^+^Th cells are essential for CD8^+^Tc effector function and antitumor immunity (5, 6).

Numerous studies have shown that the continuous antigenic stimulation of tumors *in vivo* may cause CD4^+^Th exhaustion and senescence, which are related to the occurrence and progression of cancer (7, 8). Currently, research on the relationship between CD4^+^Th cells and breast cancer is primarily focused on local immune responses in the tumor microenvironment (9, 10). However, as demonstrated in oropharyngeal cancer (11), colorectal cancer (12), and other malignancies, circulating CD4^+^Th subsets may provide potentially significant information regarding the development of malignancy. Therefore, it is of great clinical significance to understand the characteristics of circulating CD4^+^Th cells in patients with breast cancer.

Currently, surgery is the primary treatment for most patients with breast cancer that has not metastasized to distant organs (13). Complete surgical excision with clear margins can decrease a patient’s tumor burden and support the recovery of the patient’s immune system. Yu et al. demonstrated that the number of CD4^+^, CD8^+^, CD3^+^, and natural killer cells increased after hepatocellular carcinoma surgery, and immune function continued to improve (14). Wu et al. found that regulatory T cells (Tregs), which suppress the immune response, decreased in number after ovarian cancer surgery (15). In addition, studies have shown that the increased peripheral Tregs in the short term (<72 h) after surgery is associated with poor prognosis in patients with breast cancer (16). Therefore, understanding the effects of surgery on the immune system in these patients may be useful for prognostic stratification.

The proportion of CD4^+^Th cell subsets reflects the level of immune cell development and differentiation, whereas the absolute number reflects the level of immune cell proliferation. In addition, the expression of CD57 and PD-1 on CD4^+^Th cells represents immunosenescence and immune exhaustion, respectively. In this study, we evaluated the characteristics and postoperative dynamic changes in circulating CD4^+^Th cell subsets in patients with breast cancer.

## 2 Materials and methods

### 2.1 Study population

This study included 128 individuals admitted to Dongyang People’s Hospital between September 2021 and August 2022. Based on the pathological analysis of breast tissue sections, all patients were divided into two groups: those with invasive breast cancer and those with benign tumors. Patients with ductal carcinoma *in situ* and tumors that were premalignant were not included in the study. Participants had to satisfy the following inclusion criteria (1): no other primary tumors (2); no adjuvant chemoradiotherapy prior to preoperative blood sample collection (3); no apparent signs of infection; and (4) no systemic diseases such as autoimmune and blood diseases. In addition, based on the inclusion criteria, patients with invasive breast cancer who underwent radical mastectomy or modified radical mastectomy without significant postoperative complications were included in the postoperative group and followed up for 2–3 weeks after surgery.

This study was approved by the Ethics Committee of Dongyang People’s Hospital (approval no.: 2021-YX-091). All patients enrolled in the study signed informed consent.

### 2.2 Sample collection

Preoperative fresh whole blood samples (2 mL) were collected in ethylenediaminetetraacetic acid anticoagulation tubes to study the characteristics of circulating CD4^+^Th cell subsets in patients with breast cancer. For patients with invasive breast cancer who underwent postoperative follow-up, 2 mL of fasting whole blood was collected again at 2–3 weeks after surgery to evaluate the dynamic changes in CD4^+^Th cell subsets upon surgery. Samples were examined within 24 h of collection.

### 2.3 Flow cytometry

To detect immune cell surface antigens, 10-Color flow cytometry was used. In brief, the standard assay procedure was as follows (1): 100 µL of whole blood was mixed thoroughly with pre-mixed antibody (CD45RA-fluorescein isothiocyanate, clone ALB11, Beckman Coulter; CD4-phycoerythrin, clone 13B8.2, Beckman Coulter; CD28-phycoerythrin-Texas, clone CD28.2, Beckman Coulter; PD-1-phycoerythrin-cyanin 5.5, clone PD1.3, Beckman Coulter; CD27-phycoerythrin-cyanin 7, clone 1A4CD27, Beckman Coulter; CCR7-allophycocyanin, clone G043H7, BioLegend; CD8-allophycocyanin-Alexa Fluor 700, clone B9.11, Beckman Coulter; CD3-allophycocyanin-Alexa Fluor 750, clone UCHT1, Beckman Coulter; CD57-pacific blue, clone NC1, Beckman Coulter; CD45-Krome Orange, clone J.33, Beckman Coulter) and left to stand for 15 min (2); commercial red blood cell lysate (OptiLyse C Lysing Solution, Beckman Coulter) was added for complete lysis of red blood cells (3); phosphate buffer solution was added for washing, the supernatant was removed after centrifugation, and then phosphate buffer solution was added for resuspension; and (4) flow cytometry results were analyzed using the accompanying software (version 2.0, Beckman Coulter). The gating strategy of CD4^+^Th subsets is shown in **Supplementary Figure 1**.

CD3^+^CD4^+^CD8^-^T cells were defined as CD4^+^Th. CD4^+^Th was divided into the following subgroups according to its effector memory differentiation status: CD4^+^ naive T cells (Tn) (CD45RA^+^CCR7^+^CD28^+^CD27^+^), CD4^+^ central memory T cells (Tcm) (CD45RA^−^CCR7^+^CD28^+^CD27^+/−^), and CD4^+^ effector memory T cells (Tem) (CD45RA^−^CCR7^-^CD28^+/−^CD27^+/−^). The proportion and absolute number of CD4^+^PD-1^+^T cells represent the exhaustion state of CD4^+^Th cells, and those of CD4^+^CD57^+^T cells represent the senescence state of CD4^+^Th cells.

### 2.4 Clinicopathological results

The following criteria were used to determine positive immunohistochemistry: estrogen receptor (ER)/progesterone receptor (PR) positivity, the proportion of tumor nuclear stained ≥ 25% and/or intensity of staining ≥ 1+; C-erbB-2 positivity, immunohistochemical staining intensity ≥ 3+ and/or positive fluorescence *in situ* hybridization.

### 2.5 Statistical analysis

All statistical analyses of this study were conducted using IBM SPSS Statistics software (version 23.0). Categorical variables are presented as quantities (percentages). According to normal or non-normal distribution, continuous variables are presented as mean ± standard deviation or median (interquartile range). The differences between groups were compared using t-test for normally distributed variables and Wilcoxon rank-sum test for non-normally distributed variables. Postoperative dynamic changes in CD4^+^Th subsets were assessed using the paired-sample rank-sum test.

## 3 Results

Based on the inclusion criteria, 75 patients with invasive breast cancer and 53 patients with benign tumors were enrolled in the study. All patients included in the study were women. There was no significant difference in age between the breast cancer and benign tumor groups (51.7 ± 11.0 *vs*. 48.2 ± 8.9, *P* = 0.060). Of the 75 patients with invasive breast cancer, 35 were in the postoperative group. **Table 1** shows the basic characteristics of the participants included in the study.

**Table 1:** Basic characteristics of the participants included in the study.

	Benign control (N = 53)	Breast cancer (before surgery) (N = 75)	Breast cancer (after surgery) (N = 35)
Age, years	48.2 ± 8.9	51.7 ± 11.0	48.7 ± 10.1
Stage, n (%)			
I		32 (42.7)	11 (31.4)
II		27 (36.0)	16 (45.7)
III		12 (16.0)	8 (22.9)
IV		4 (5.3)	0 (0.0)
Estrogen receptor status, n (%)			
Positive		56 (74.7)	21 (60.0)
Negative		19 (25.3)	14 (40.0)
Progesterone receptor status, n (%)			
Positive		52 (69.3)	18 (51.4)
Negative		23 (30.7)	17 (48.6)
C-erbB-2 status, n (%)			
Positive		18 (24.0)	11 (31.4)
Negative		57 (76.0)	24 (68.6)

### 3.1 Preoperative CD4^+^Th subsets in the breast cancer and benign tumor groups

As shown in **Table 2**, compared with that in patients with benign tumors, the proportion of CD4^+^Tn cells in patients with breast cancer decreased (mean: 27.9 *vs*. 33.5, *P* = 0.011), while the absolute number and proportion of CD4^+^CD57^+^T and CD4^+^PD-1^+^T cells significantly increased (*P* < 0.05).

**Table 2:** Preoperative CD4^+^Th subsets in the breast cancer and benign tumor groups.

	Benign control (N = 53)	Breast cancer (before surgery) (N = 75)	*P*-value
CD4^+^Th, % of T cells	54.5 ± 9.4	54.0 ± 10.1	0.802
CD4^+^Tn, % of CD4^+^Th	33.5 ± 13.3	27.9 ± 11.1	0.011
CD4^+^Tcm, % of CD4^+^Th	38.2 ± 7.8	40.0 ± 9.0	0.221
CD4^+^Tem, % of CD4^+^Th	26.0 (18.5–34.7)	29.3 (23.4–38.1)	0.089
CD4^+^CD57^+^T, % of CD4^+^Th	4.2 (2.7–6.2)	6.6 (3.5–10.2)	0.002
CD4^+^PD-1^+^T, % of CD4^+^Th	34.6 ± 8.2	39.3 ± 10.5	0.008
CD4^+^Th, 10^6^/L	551.0 (497.0–771.1)	617.8 (497.3–725.2)	0.560
CD4^+^Tn, 10^6^/L	187.5 (135.4–260.6)	176.3 (111.7–226.1)	0.160
CD4^+^Tcm, 10^6^/L	219.1 (182.4–269.4)	247.5 (197.0–299.3)	0.137
CD4^+^Tem, 10^6^/L	168.1 (117.1–203.5)	183.1 (133.9–230.0)	0.060
CD4^+^CD57^+^T, 10^6^/L	23.9 (15.6–41.7)	41.5 (22.1–57.7)	0.001
CD4^+^PD-1^+^T, 10^6^/L	198.4 (172.1–241.0)	222.7 (181.5–280.1)	0.024

Tem, effector memory T cells; Tn, naive T cells; Tcm, central memory T cells; Th, helper T cell.

### 3.2 Association between CD4^+^Th and clinicopathology

**Figure 1:**
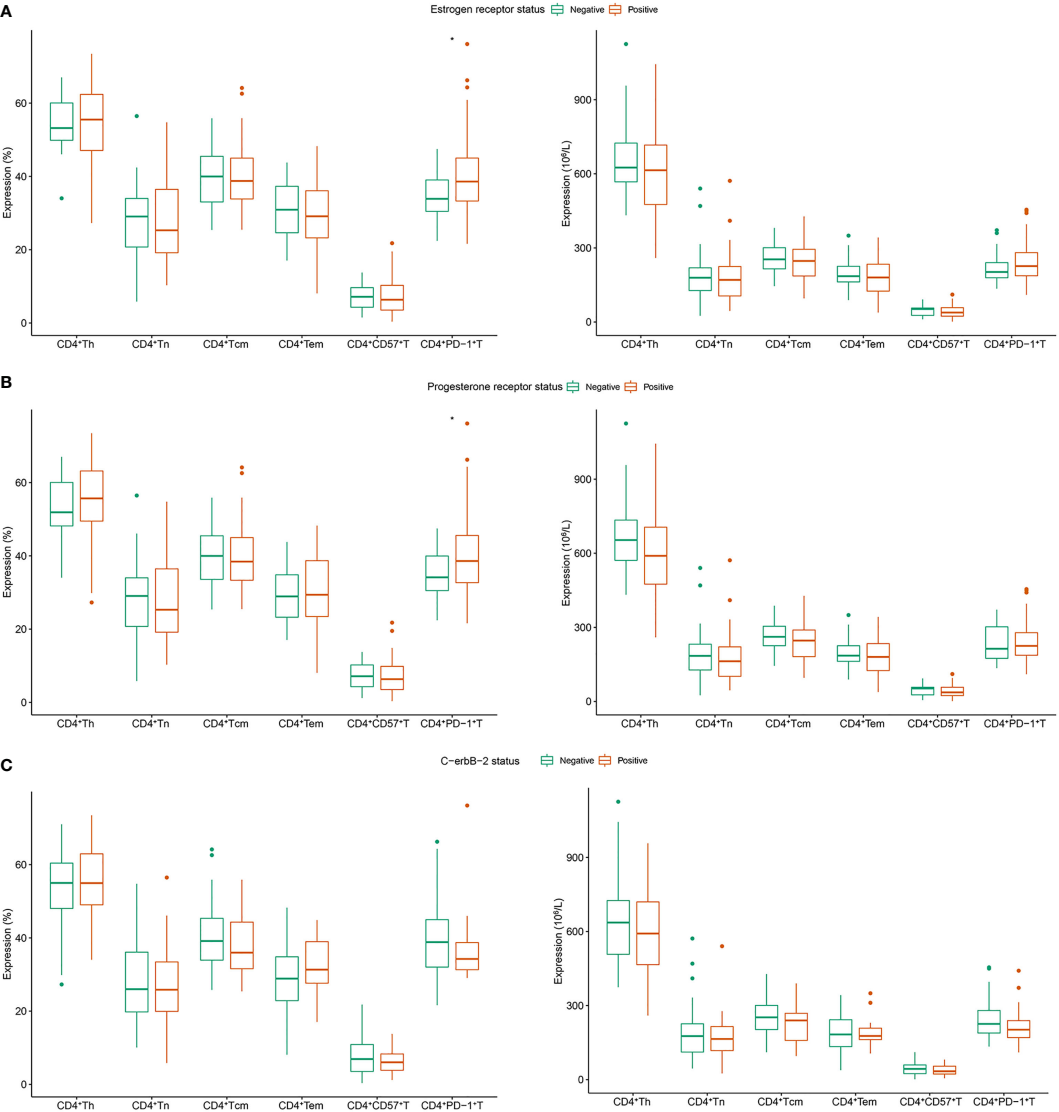
Correlation between CD4^+^Th subsets and clinicopathology in patients with breast cancer. **(A)** Estrogen receptor; **(B)** progesterone receptor; **(C)** c-erbB-2. Tn, naive T cells; Tem, effector memory T cells; Tcm, central memory T cells; Th, helper T cell.

In patients with positive and negative ER expression, the median CD4^+^PD-1^+^T (% of CD4^+^Th) population was 38.6% and 33.9%, respectively (*P* = 0.029, **Figure 1A**). Similarly, patients with positive PR expression had higher levels of CD4^+^PD-1^+^T (% of CD4^+^Th) than those with negative PR expression (median: 38.6% *vs*. 34.10%, *P* = 0.032, **Figure 1B**). No relationship existed between the proportion or absolute number of CD4^+^Th subsets and C-erbB-2 expression (**Figure 1C**).

### 3.3 Postoperative dynamic changes in circulating CD4^+^Th cells in patients with breast cancer

**Figure 2:**
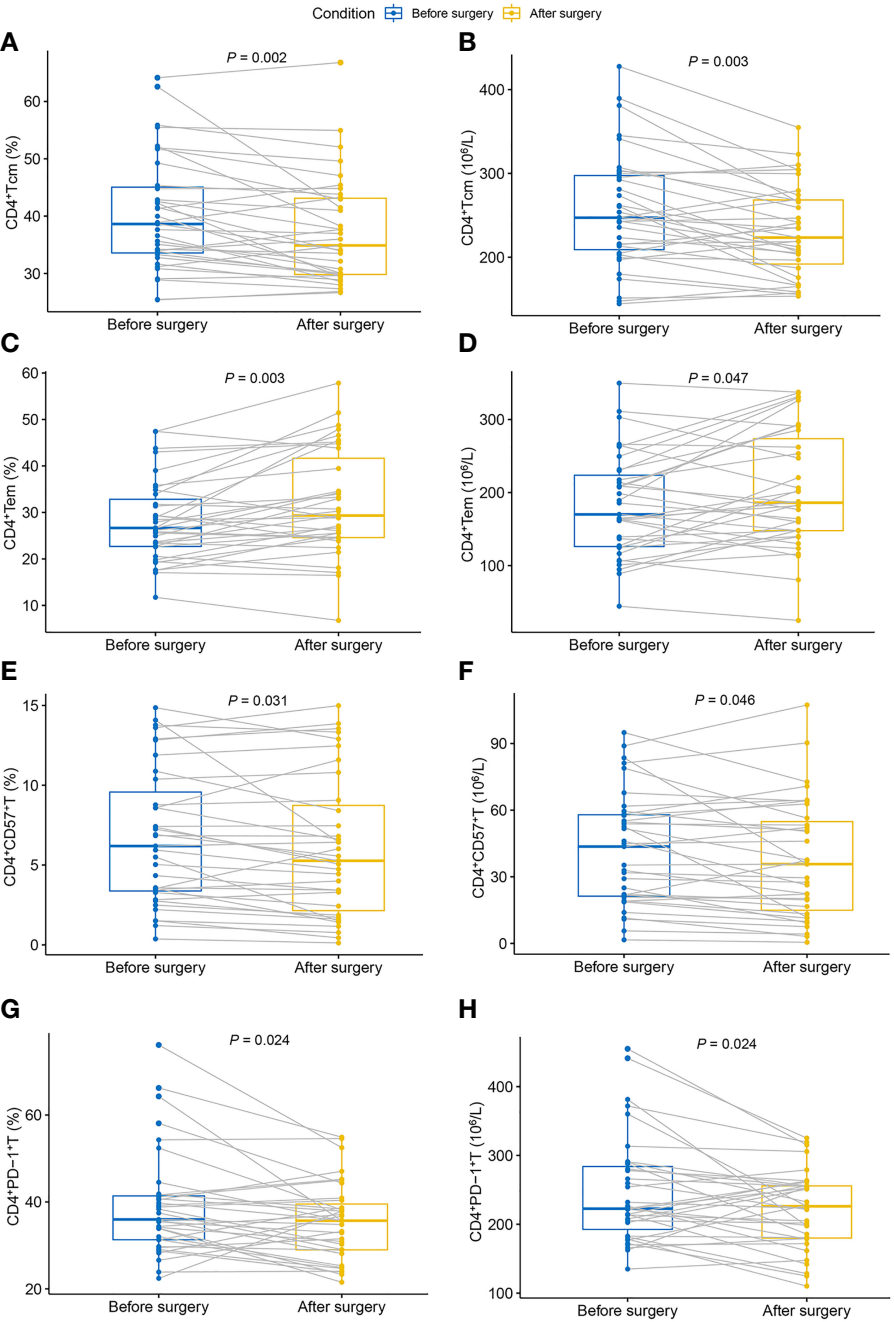
Dynamic changes in circulating CD4^+^Th cells in patients with breast cancer after surgery. **(A)** Proportion of CD4^+^Tcm; **(B)** absolute count of CD4^+^Tcm; **(C)** proportion of CD4^+^Tem; **(D)** absolute count of CD4^+^Tem; **(E)** proportion of CD4^+^CD57^+^T; **(F)** absolute count of CD4^+^CD57^+^T; **(G)** proportion of CD4^+^PD-1^+^T; **(H)** absolute count of CD4^+^PD-1^+^T. Tem, effector memory T cells; Tcm, central memory T cells; Th, helper T cell.

**Table 3** shows the dynamic changes in circulating CD4^+^Th cells before and after surgery. After tumor resection, the proportion and absolute number of CD4^+^Tcm decreased significantly compared with the preoperative values (**Figures 2A, B**). Moreover, the proportion and absolute number of CD4^+^Tem significantly increased after tumor resection (**Figures 2C, D**). Notably, tumor resection caused significant changes in the proportion and absolute number of CD4^+^CD57^+^T and CD4^+^PD-1^+^T, both of which showed a significant decrease (**Figures 2E, H**).

**Table 3:** Dynamic changes in CD4^+^Th subsets in patients with breast cancer before and after surgery.

	Breast cancer (before surgery) (N = 35)	Breast cancer (after surgery) (N = 35)	*P*-value
CD4^+^Th, % of T cells	54.6 ± 9.4	55.0 ± 10.2	0.967
CD4^+^Tn, % of CD4^+^Th	29.9 ± 11.3	29.1 ± 11.2	0.064
CD4^+^Tcm, % of CD4^+^Th	40.3 ± 10.0	37.3 ± 9.3	0.002
CD4^+^Tem, % of CD4^+^Th	28.1 ± 8.7	32.0 ± 11.5	0.003
CD4^+^CD57^+^T, % of CD4^+^Th	6.7 ± 4.3	6.1 ± 4.4	0.031
CD4^+^PD-1^+^T, % of CD4^+^Th	39.2 ± 12.2	35.6 ± 8.9	0.024
CD4^+^Th, 10^6^/L	645.7 ± 150.1	632.9 ± 125.6	0.578
CD4^+^Tn, 10^6^/L	197.2 ± 102.3	188.3 ± 91.8	0.201
CD4^+^Tcm, 10^6^/L	255.5 ± 68.2	231.1 ± 54.0	0.003
CD4^+^Tem, 10^6^/L	182.7 ± 70.0	203.7 ± 80.6	0.047
CD4^+^CD57^+^T, 10^6^/L	42.2 ± 25.3	37.4 ± 26.5	0.046
CD4^+^PD-1^+^T, 10^6^/L	247.5 ± 78.0	221.6 ± 56.6	0.024

Th, helper T cell; Tn, naive T cells; Tcm, central memory T cells; Tem, effector memory T cells.

## 4 Discussion

At present, none of the new blood markers for the diagnosis of breast cancer, such as circulating tumor cells (17) and microRNA (18, 19), has reached the standard of clinical routine practice, while circulating immune cells are considered a potential key breakthrough. This study found that the proportion of CD4^+^Tn cells was decreased in patients with breast cancer, while the absolute number and proportion of CD4^+^CD57^+^T and CD4^+^ PD-1^+^T cells were significantly increased compared with those in patients with benign tumors. Moreover, the proportion of CD4^+^PD-1^+^T was correlated with the clinicopathology of breast cancer. Notably, this study is the first to report significant changes in the proportion and number of circulating CD4^+^Th cell subsets after surgical resection of tumors.

### 4.1 Distribution of CD4^+^Th cells from naive to effector memory in patients with breast cancer

We observed that the proportion of CD4^+^Tn cells in breast cancer patients decreased. A similar distribution was previously found for head and neck cancer (20). The decrease in Tn, a newly formed reserve cell, suggests that the immune system’s reserves of CD4^+^T cells are depleted after prolonged activation by cancer antigens.

Antigen-specific CD4 persisted in Tcm and Tem cells. The functional expression of these two subsets is apparently different from one another, with the latter directly influencing patient prognoses. Tada et al. showed that low CD4^+^Tem level is a poor prognostic factor in patients with colorectal cancer (21). The current study found that the proportion and absolute number of CD4^+^Tcm cells decreased and that of CD4^+^Tem cells increased after surgery. This phenomenon of postoperative immune activation has also been reported in pancreatic cancer (22). For patients without distant metastasis, the decreased tumor burden can reverse the immunosuppressive status and improve patient prognosis.

### 4.2 Expression of PD-1 in patients with breast cancer

PD-1 (CD279) is commonly used to assess T cell exhaustion as it negatively regulates T cell proliferation and cytokine production, resulting in dysregulation of host immunity (23). Zhu et al. found that CD4^+^PD-1^+^T cells in the peripheral blood of patients with thyroid cancer had a higher count than those of patients with nodular goiter (24). Furthermore, Rosenblatt et al. demonstrated a significant increase in PD-1 expression in circulating CD4^+^Th cell populations in patients with active myeloma (25). In our study, we found that the proportion and absolute number of CD4^+^PD-1^+^T cells in patients with breast cancer were higher than those in patients with benign tumors, and the proportion of CD4^+^PD-1^+^T was related to clinicopathology.

Numerous studies have shown that PD-1 expression is closely related to treatment response in patients with cancer (26, 27). However, some studies have reported that the prognostic value of PD-1 expression is uncertain because of the presence of high tumor heterogeneity, which is affected by the choice of detection method and antibody (28). At present, PD-1 detection is mostly done through the tumor tissue samples’ immunohistochemical analysis, which is not convenient for follow-up monitoring (29). In this study, circulating CD4^+^PD-1^+^T cells were detected with flow cytometry, and it was found that surgical resection of the tumor could reverse the immune exhaustion status of patients with breast cancer.

### 4.3 Expression of CD57 in patients with breast cancer

In the occurrence and development of breast cancer, in addition to T cell exhaustion, T cell senescence also produces malignant positive feedback. T cell exhaustion and senescence have similar manifestations but completely different origins. In contrast to exhaustion, which is controlled by extrinsic immunomodulatory mechanisms, immunosenescence is controlled in nature by the intrinsic stress response of immune cells (30). Studies have shown that CD57 is the most relevant marker of T cell senescence because the proliferation ability of CD57-expressing T cells is severely impaired in cancer (31). Shiraki et al. demonstrated that the percentage of CD4^+^CD57^+^T cells in the peripheral blood of patients with hepatitis C virus-associated hepatocellular carcinoma increased with tumor progression (32). Similar results can be seen in other malignancies, such as oral squamous cell carcinoma (33) and gastric cancer (34). In this study, the proportion and number of circulating CD4^+^CD57^+^ T cells in patients with breast cancer increased, and immunosenescence occurred.

Immunosenescence has long been considered irreversible. However, Beausejour et al. showed that immunosenescence may not necessarily be permanent (35). Moreover, Fornara et al. demonstrated a sustained decrease in the proportion of CD4^+^CD57^+^ T cells after surgery in patients who were long-term glioblastoma survivors (36). This study found that patients without distant metastasis showed a decreased CD4^+^CD57^+^ T cell proportion and absolute number after surgery, thus reversing immunosenescence.

This study describes the characteristics of circulating CD4^+^Th cells in patients with breast cancer and demonstrates for the first time that surgical treatment of breast cancer creates a new balance between immune suppression and immune stress in patients. However, this study has some limitations. First, prognostic information in patients with breast cancer cannot be obtained directly from this study. Therefore, more research is required to determine whether dynamic changes in CD4^+^Th influence the survival rate of patients. Additionally, the limited sample size from a single center limits the generalizability of results, requiring further validation.

In conclusion, this study found significant changes in circulating CD4^+^Th subsets in patients with breast cancer, which were related to clinicopathology. In addition, complete surgical resection of tumors can benefit patients, which can create a new balance between immunosuppression and immune activation, and reverse the state of immune exhaustion and immunosenescence.

## Data availability statement

The original contributions presented in the study are included in the article/**Supplementary Material**. Further inquiries can be directed to the corresponding author.

## Ethics statement

The studies involving human participants were reviewed and approved by the Ethics Committee of Dongyang People’s Hospital (approval: 2021-YX-091). The patients/participants provided their written informed consent to participate in this study.

## Author contributions

All authors contributed to the study conception and design. Material preparation and data collection and analysis were performed by JW and LZ. The first draft of the manuscript was written by YL and QZ. All authors commented on previous versions of the manuscript. All authors contributed to the article and approved the submitted version.
