# Supplementary material for: Characteristics and postoperative dynamic changes in circulating CD4+ helper T lymphocytes in patients with breast cancer
Source: Front Oncol. 2023 Feb 28;13:1118346. doi: 10.3389/fonc.2023.1118346 (PMC10011473; doi:10.3389/fonc.2023.1118346)
Supplement: Supplementary file 1 [file Image_1.pdf]

Supplementary Material

Characteristics and postoperative dynamic changes in circulating CD4$^+$ helper T lymphocytes in patients with breast cancer

Yan Lu, Qiaohong Zhang, Jiang Wang, Longyi Zhang*

*** Correspondence:** Longyi Zhang

happy_zhang1y@163.com

1 Supplementary Figure

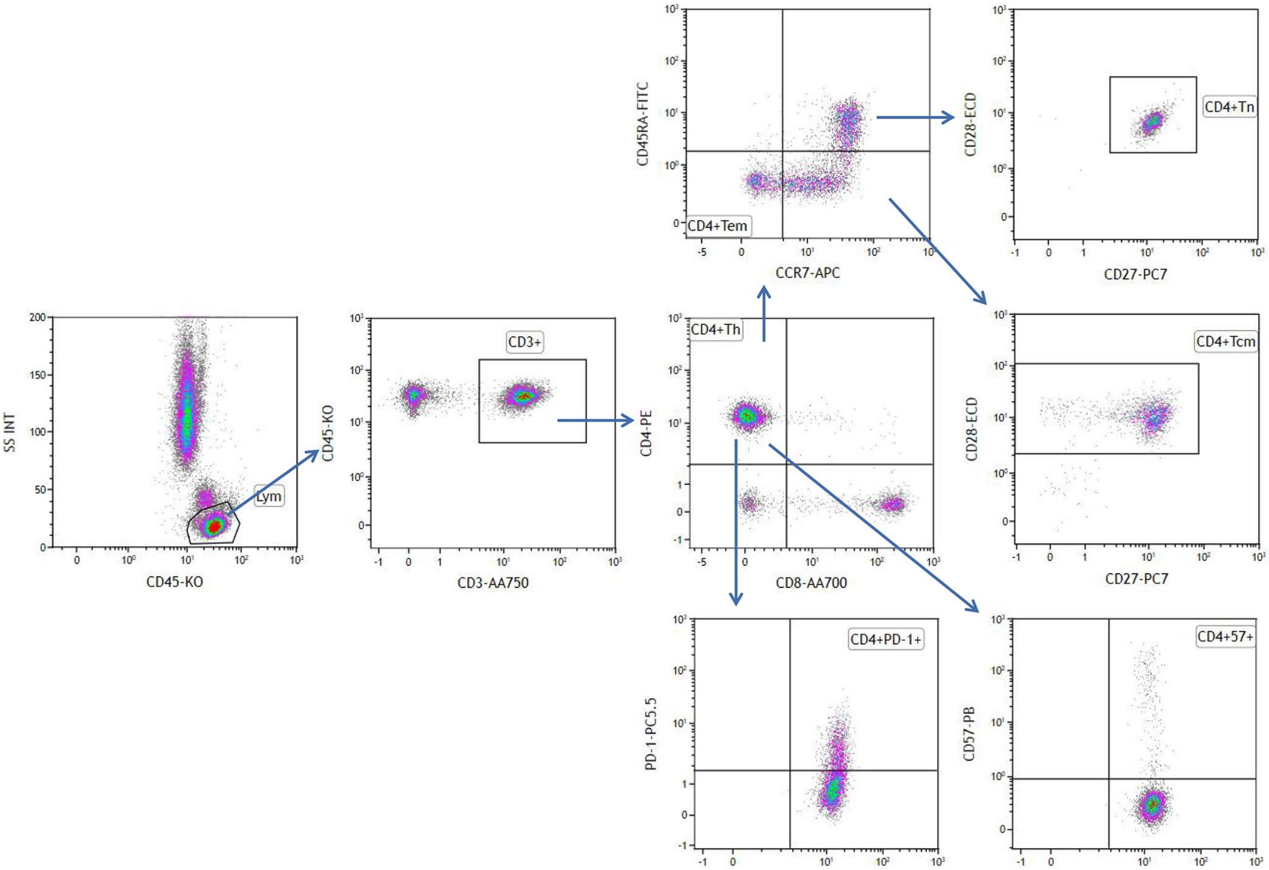

Supplementary Figure 1. The gating strategy of CD4^{+}Th subsets.
